# Sensor Selection Framework for Designing Fault Diagnostics System

**DOI:** 10.3390/s21196470

**Published:** 2021-09-28

**Authors:** Amol Kulkarni, Janis Terpenny, Vittaldas Prabhu

**Affiliations:** 1 Department of Industrial and Manufacturing Engineering, The Pennsylvania State University, State College, PA 16801, USA; amolkulkarni@psu.edu (A.K.); vittal.prabhu@psu.edu (V.P.); 2 Department of Industrial and Systems Engineering, The University of Tennessee, Knoxville, TN 37996, USA

**Keywords:** sensor selection, fuzzy clustering, ordered clustering

## Abstract

In a world of rapidly changing technologies, reliance on complex engineered systems has become substantial. Interactions associated with such systems as well as associated manufacturing processes also continue to evolve and grow in complexity. Consider how the complexity of manufacturing processes makes engineered systems vulnerable to cascading and escalating failures; truly a highly complex and evolving system of systems. Maintaining quality and reliability requires considerations during product development, manufacturing processes, and more. Monitoring the health of the complex system while in operation/use is imperative. These considerations have compelled designers to explore fault-mechanism models and to develop corresponding countermeasures. Increasingly, there has been a reliance on embedded sensors to aid in prognosticating failures, to reduce downtime, during manufacture and system operation. However, the accuracy of estimating the remaining useful life of the system is highly dependent on the quality of the data obtained. This can be enhanced by increasing the number of sensors used, according to information theory. However, adding sensors increases total costs with the cost of the sensors and the costs associated with information-gathering procedures. Determining the optimal number of sensors, associated operating and data acquisition costs, and sensor-configuration are nontrivial. It is also imperative to avoid redundant information due to the presence of additional sensors and the efficient display of information to the decision-maker. Therefore, it is necessary to select a subset of sensors that not only reduce the cost but are also informative. While progress has been made in the sensor selection process, it is limited to either the type of the sensor, number of sensors or both. Such approaches do not address specifications of the required sensors which are integral to the sensor selection process. This paper addresses these shortcomings through a new method, OFCCaTS, to avoid the increased cost associated with health monitoring and to improve its accuracy. The proposed method utilizes a scalable multi-objective framework for sensor selection to maximize fault detection rate while minimizing the total cost of sensors. A wind turbine gearbox is considered to demonstrate the efficacy of the proposed framework.

## 1. Introduction

The performance of every system degrades over time due to external factors such as the environment it operates in or due to its operating condition. Maintenance is the key to ensuring the safe and reliable operation of a system throughout its operational life. Depending on the industry, ineffective maintenance could cost the industry up to $60 billion each year [1]. With the development of the concept of the internet of things (IoT) and the development of wireless sensor network technology, a newer maintenance strategy termed Prognostics and Health Monitoring (PHM) is growing in usage due to its cost-effectiveness and the increasing availability of the number of Internet-enabled devices on the market [2]. The functional architecture of PHM typically consists of six layers, including 1. Data acquisition 2. Data Manipulation 3. Condition Monitoring 4. Health assessment 5. Prognostics and 6. Decision Making.

Since the data acquisition layer forms the bedrock on which subsequent layers depend, it is imperative to provide high-quality information for effective fault diagnosis and prognosis to aid in decision making. A primary component of the data acquisition layer is the sensor units. Sensor units provide a means for measuring, monitoring and tracking environmental and operational parameters. Considering the recent developments in sensor technology there are numerous types of sensors available in the market to measure parameters such as displacement, acceleration, force, temperature, light, touch, location, gas and biological matter [3]. In light of the increasing type and number of sensors, for effective deployment of the PHM system, an efficient sensor selection process needs to be established. (in order to avoid confusion, it should be noted that sensor selection has two meanings in the field. One refers to the selection of sensors from the set already deployed into a network; the selection process is used to optimize the network by choosing which sensors will be active at a given time [4]. The meaning intended in this work refers to the selection of sensors to be integrated into a system during the design and build process or after the process; in this case, the selection process is focused on incorporating the most appropriate sensors for the task at hand [5]). 

The sensor selection process varies from smaller systems or experimental setups to larger systems that are deployed and being used by the end-user. The sensor selection process for the experimental systems/setups is empirical in nature and simple, whereas the selection process for large complex systems is mainly systematic and requires several additional considerations. Several sensor selection methods have been proposed over the years, but most methods are not scalable, i.e., the selection method for experimental setups cannot be used for large complex systems and vice versa. In addition, the task of sensor selection itself is complicated by the lack of standard vocabulary in characterizing existing sensors and associated specifications. Most existing sensor selection processes are quite complicated; some require the use of specialized software and advanced approaches. This paper aims to establish a sensor selection framework suitable for both experimental setups and large complex systems.

**Figure 1:**
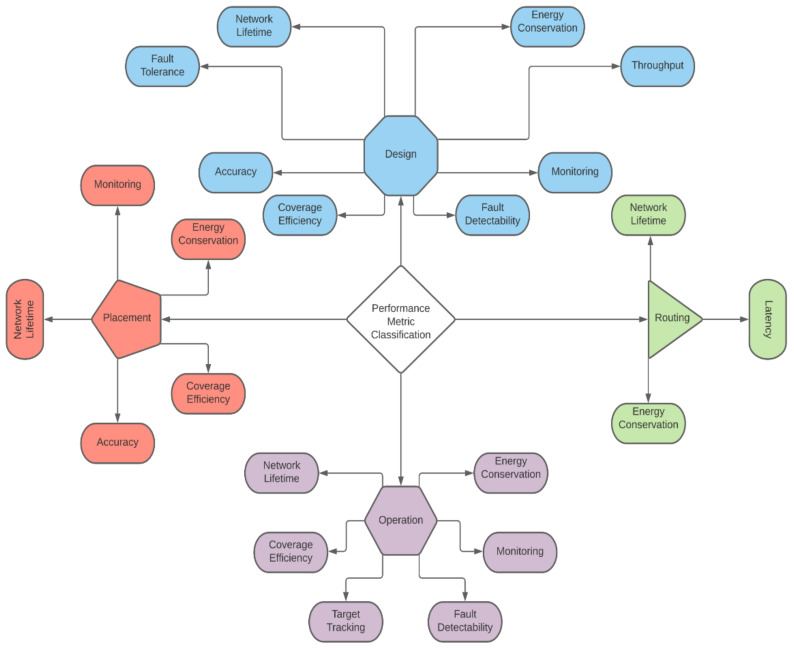
Classification of Objectives for Sensor.

In the past, sensor selection relied heavily on the domain knowledge of the decision-maker thereby making it subjective. Recently, several sensor selection methods have been proposed based on graphical and semantic approaches, with explicitly stated objectives and constraints as shown in Figure 1. The objectives are also referred to as performance requirements or figures-of-merits (FOM) in the literature. Some sensor selection processes have been proposed for experimental setups, but most of them are for large complex systems. Sensor selection as outlined by Santi, Sowers and Aguilar [6], forming the basis for the methods proposed in the literature. This process starts with design engineers creating a detailed and unambiguous list of the operating, environmental, physical and cost specifications for the system. After building an in-depth understanding of the physics of the system and its use context, the next step is to identify the measurement principle. Next is the identification of the sensing methods. Note that a multitude of sensing methods are available for each of the candidate measurement principles in the list. The final step in the process is to select the sensors that best fit the constraints of the scenario. In Cheng, Azarian and Pecht [7], the authors discuss criteria such as parameters to be measured, performance needs, electrical and physical attributes, reliability and cost for sensor system selection for PHM applications. In the next section, a review of the literature associated with the study of sensor selection is classified into (a) empirical sensor selection process for experimental setups and (b) sensor selection through structural equations for large complex systems.

### 1.1. Empirical Sensor Selection Process

A graphical toolset for sensor selection using 2D performance charts has been proposed [8]. This process focuses on collecting and defining the sensor characteristics for each sensor type from the manufacturers’ datasheets. The collected information is then plotted on two-dimensional charts, with sensor performance indices as the axes. These performance charts illustrate various trade-offs; for example, resolution vs. range or frequency vs. range. Only the common elements corresponding to a type of sensor listed on the manufacturers’ technical specifications are used for the performance charts. The strongest sensor candidates are identified from the charts. A subset of the sensor candidates is then selected after accounting for cost, practicality and reliability. According to the authors, this method gives an overview of sensor performance thus graphically illustrating the sensors best suited for a given task. Although this method acts as a straightforward visual selection tool, it is not scalable. Unstructured and variable outcomes result due to the lack of a systematic method to guide the designers in the pairing of attributes within the performance chart. 

Schmidt and Laerhoven [9] proposed a semantic approach to sensor selection in the context of building a smart appliance. The approach begins with the analysis of the conditions of the informational, physical and social environment in which the appliance is used or interacted with. Situations that are similar for the device are grouped in a single context that is labeled. Variables such as time interval, temperature, value, number of people in the vicinity, etc. that help discriminate the contexts are identified. Based on the variables identified, sensors are selected while accounting for the accuracy and cost of the sensors. The selection begins all over if the sensors do not perform well in the lab setting with a prototype of the device. This is time-consuming and an inefficient approach for sensor selection. A similar context-aware approach to sensor selection using the dynamic skyline technique is proposed by Kertiou et al. [10]. The dynamic skyline technique is utilized to reduce the search space and select the best sensors following user requirements. According to the authors, this method can be adopted by different IoT middleware for designing relevant solutions with a high level of accuracy and minimize the search and selection time. To counter the slow-acting dynamic skyline technique and to make it scalable the authors propose the use of distributed gateways connected to a server, each gateway responding to a local request by the user. 

A novel sensor selection algorithm utilizing the concept of entropy and information gain from information theory is proposed by Tjen, Smarra and D’Innocenzo [11] for structural damage detection. The main idea is to choose a sensor from each pair of sensors such that the information gain is maximized. The authors use a Principal Component Analysis-based metric to achieve a trade-off between prediction accuracy and computational complexity. Zhang, Ayoub and Sundaram [12] show that greedy algorithms are optimal for estimating the states for a certain class of linear dynamical systems. Along with budget constraints, they consider the objective of minimizing the trace of the steady-state a priori or a posteriori error covariance produced by a Kalman filter. The authors also provide proof that even under the assumption of a stable system, a priori and a posteriori error covariance-based sensor selection problems are NP-hard. The authors also demonstrate that certain objective functions are not submodular or supermodular in general which makes it difficult to evaluate the performance of greedy algorithms in theory. Through simulations, the performance of the proposed greedy algorithms is illustrated. A similar greedy algorithm approach is proposed by Clark, Brunton and Kutz [13] to approximate the number of economic and expensive sensors in an environment or state space. The authors evaluate the composition of both types of sensors along with their placement to assess their ability to reconstruct a higher dimensional state space. The preliminary sensor positions are obtained through QR-decomposition. The sensor noise levels, sensor cost, total budget and the single value spectrum of the data measured play a significant role in selecting the number of sensors. The sensor recommendations are based on the computational results of asymptomatic regions of parameter space.

A three-sieve sensor selection method is proposed by Jones et al. [14] which takes into account performance requirements, environmental constraints and economic considerations. This method starts with an analysis of the system. The candidate sensors are assessed for specific requirements from the operators and the final decision is based on the cost of the sensors. This method, however, is meant specifically for experimental setup and small systems. This method can be used to consider homogenous sensors and the comparison can be made only with similar sensors.

### 1.2. Sensor Selection through Linear and Non-Linear Equation

To guide the sensor selection process in highly complex systems with a large number of interacting parameters, modeling tools and software may be needed. The Drexel University Intelligent Infrastructure Institute proposed a sensor selection methodology for bridge health monitoring [15]. The first step in the process is to analyze the bridge and its surroundings as well as the environment in which the sensors are needed to be deployed. Based on the analysis a candidate set of sensors are selected in consideration of performance characteristics, environmental constraints and cost. Zhang and Vachtsevanos [16] proposed a methodology to decide the type, number and location of sensors. A novel graph-based technique called quantified-directed-model for fault propagation from subsystems to subsystems in a large complex system is presented. The authors quantify the fault detectability metric via signal-to-noise ratio, time-to-detection to the time-to-failure ratio, sensitivity of a sensor and symptom duration to time-to-failure ratio. These, along with cost as the objectives, are modeled and optimized using particle swarm optimization. The performance of the proposed method is tested on a five-tank system.

A knowledge-based selection of sensors and actuators for plant equipment was proposed by Riedel, Arroyo and Fay [17]. The authors argue that selection decisions are taken one device at a time, which is time-consuming and results in suboptimal solutions. To overcome this limitation the proposed method presents a concept based on plant description and semantic models. The paper states that this function-oriented selection process is capable of considering a wider solution space as well as seamless integration of this procedure into plant workflow. L. Santi’s’ [6] a systematic sensor selection (S4) policy for aerospace vehicle design forms the basis for most sensor selection processes. The proposed method supports the selection of sensors adapted to a system in a particular situation. After establishing constraints through a computer-assisted analysis, sensor selection is carried out via a process of iterative optimization. The proposed method addresses a complex situation that needs a large number of interacting sensors. This method is also utilized for boost stage rocket engines, turbo-fan engine diagnostics, and aircraft engine health estimation [18,19,20]. A sensor selection based on the physical model and sensitivity analysis for a helicopter transmission system is proposed by Lyu et al. [21]. The first step in the proposed method builds a physical model of the gear tooth damage and mesh stiffness. In the next step, effective condition indicators (CI) are presented and the optimal CI set is selected via the Mann-Kendall test. The selected optimal CI is used to develop a health indicator through the sen slope estimator. Based on the monotonic relevance and sensitivity to damage levels sensors are selected. The selected approach is validated by simulation. The authors state that the proposed approach effectively reduces the test cost and improves the system’s reliability. The proposed methods require knowledge of advanced modeling software and algorithms which can time consuming and expensive to implement making it viable only for large complex systems.

Based on sensitivity analysis and the capability of the sensors in predicting the polymer electrolyte membrane (PEM) fuel cell performance sensor selection algorithms such as the largest gap method and exhaustive brute force search are explored by Mao and Jackson [22]. A sensitivity matrix related to sensor measurements and fuel cell health parameters is generated using a fuel cell model. The sensitivity matrix is used as the input for the sensor selection algorithms proposed in the paper. The authors demonstrate that accurate prediction can be obtained with optimal sensors. A sensor selection algorithm for PEM fuel cells considering sensor sensitivity, fuel cell performance and resistance to noise is proposed by Mao, Davies and Jackson [23]. The sensitivity of the sensors is calculated via a fuel cell model and the sensitivity to different failure modes is then ranked. The performance of the selected sensors is evaluated via an adaptive neuro-fuzzy inference system (ANFIS). The proposed methods are focused mainly on the health of PEM fuel cells; it is not suitable to be used as a general tool for sensor selection.

A comprehensive evaluation method of sensor selection for PHM based on grey clustering for an electronic control system of radar was proposed by Guan et al. [24]. The first step in the proposed approach is to define and quantify three grey indexes based on the dependency matrix and classify the sensors into grey classes. The next step is to utilize the whitening weight function in consideration of the objective and subjective tendency to improve the effectiveness of the result. The final step in the process is to cluster the sensors by analyzing the clustering coefficient calculated based on grey clustering theory.

The most commonly used sensors for predictive maintenance of industrial motors are listed by Murphy [25]. The report summarizes the advantages and disadvantages of various sensors and when to use sensors for health monitoring. It also acts as a guide for parameters that need to be sensed/measured for predictive maintenance. However, beyond simple comparison, it does not provide a method for selecting the sensors. Summary of the literature review is shown in Table 1.

Based on the literature review the following conclusions can be drawn. First, the sensor selection process regardless of the application technology always begins with the analysis of the system and its failure modes. Second, the selected sensors need to be compared with the specific system and environmental constraints. Third, the sensor selection tools developed in the literature for both classes of the sensor selection process are not interchangeable. Finally, the cost considerations are taken into account after considering the technical constraints. Additionally, the methods proposed are not usable if the practitioner is not well versed in semantic processes or complex selection algorithms.

## 2. An Ordered Fuzzy Clustering Approach to Sensor Selection

This section introduces the OFCCaTS (Ordered Fuzzy C-means Clustering and Two Sieve) methodology for sensor selection. This includes an overview of the methodology as well as details for each of its core components. The proposed sensor selection process integrates the features of a typical sensor selection process and facilitates making a final decision based on the system requirements. The selection process starts with the analysis of the system and assesses the sensor needs for condition monitoring and allows for the selection of the sensor right down to the specification of the sensor. The few key steps that set this sensor selection process apart are the utilization of constraints that are general to most engineered systems while also catering to the specific needs of each system with the integration of the two-sieve method. Sensors are considered clustering objects. A fault-sensor dependency matrix is created. To design an effective PHM system, the following parameters are considered: fault detection probability, fault tolerance, sensor value and fault detection time. To estimate some of these parameters, a few common assumptions are made. The first assumption is that all the sensor data are forward to a central data processing unit. This is a simple and convenient assumption as it does not require the use of any distributed computing algorithms for statistical computation. The second assumption is that the data received by the fusion center are not corrupted by any communication fault. The final assumption is that the data fusion center indicates whether the operational condition of the system is healthy or abnormal. The definitions of the parameters considered are provided below.

### 2.1. Fault Sensor Dependency Matrix

Fault-sensor mapping matrix is a diagnostic model utilized to catch the fault data and its causal relationship at the hierarchical system level [26]. It typically consists of the dependency relationship between observable failure modes and symptoms associated with a system. The fault dependency matrix is modified to reflect the relationship between the fault modes and the sensors, as the fault diagnosis here depends largely on the information collected by the sensors. A matrix $D=\left[d_{ij}\right],\;i=1,\;2,\;3,\;\ldots ,\;n$ denotes the system fault-sensor dependency matrix. If a sensor $s_{j}$ can detect the fault $f_{i}$, element $d_{ij}$ = 1; otherwise, $d_{ij}$ = 0. The fault-sensor mapping matrix is shown below:

The parameters considered below will be used to form the clustering object, which will be used as the input for the clustering algorithm.

#### 2.1.1. Likelihood Estimation

The fault-sensor mapping matrix approximately describes the simple matched relationship between fault modes and sensor set. In Table 2, $d_{ij}$ = 1 indicates that the sensor can detect the fault $f_{i}$ with a probability of 1. Due to sensor reliability and complex environmental factors, a sensor may not detect a fault with absolute certainty. The fault detection probability for each sensor is obtained via MCMC (Markov Chain Monte Carlo) simulation in python-3.

#### 2.1.2. Sensor Value Estimation

Cost is always a factor while designing any system; the same holds for PHM systems as well. To evaluate the cost of sensors usually purchase cost, installation cost, data processing cost and the sensor usage cost is considered. However, this does not accurately reflect the sensor value. To calculate the sensor value, the following parameters are considered:

Maintenance Cost = Labor Cost + Productivity Loss Cost + Component Replacement Cost,(1)

Sensor Cost = Purchase Cost + Installation Cost + Sensor Communication Cost + Sensor Replacement Cost + Disassembly Cost + Inspection Cost,(2)

(3) $$Sensor\;Value\left(i\right)=\frac{Maintenance\;Cost+Sensor\;Cost}{Total\;Number\;of\;Sensor\left(i\right)}$$

#### 2.1.3. Fault Tolerance

The reliability of a sensor after a fault has occurred is defined as fault tolerance. It is difficult to replace the sensors when they operate in extreme environmental conditions or remote places such as space. Fault tolerance depends on the application in which the sensors are deployed. Given a set of sensors *K*, the reliability of the sensors *R*(*K*, *t*) is defined as the probability that no sensor in *K* fails during the interval (0, *t*). If sensor failures are independent, one has:

(4) $$\begin{array}{c} R\left(K,t\right)=\Pi_{k\in K}R_{k}\left(t\right)\Pi_{k\notin K}\left(1-R_{k}\left(t\right)\right) \end{array}$$

where *R_k_*(*t*) is the reliability of sensor *k*. The reliability of the sensors is modeled as a Poisson distribution:

(5) $$\begin{array}{c} R_{k}\left(t\right)=e^{-\lambda_{k}t} \end{array}$$

where *λ_k_* is the failure rate of sensor *k*, typically considered to be constant under steady-state conditions. According to reliability engineering, the sum of reliability and unreliability of any component or system should be 1. Therefore, the unreliability or the probability of failure of the sensors is given by

(6) $$\begin{array}{c} Q_{k}\left(t\right)=1-R_{k}\left(t\right) \end{array}$$

#### 2.1.4. The Proportion of Fault Detection

The proportion of fault detection is defined as the ratio of the number of faults a sensor can detect to all the faults under consideration. This metric considers the proportion of faults that can be detected by the sensors. If a sensor can detect all the faults under consideration, then the value is 1. However, in reality, no one sensor can detect all the failures, and therefore the value lies between 0 and 1. The proportion of fault detection is given by the following equation.

(7) $$\begin{array}{c} PFD=\frac{\sum f_{ij}}{F}\; \end{array}$$

where,$\;f_{ij}$ is the fault ‘*i*’ that is detected by sensor ‘*j*’. F is the total number of faults under consideration.

#### 2.1.5. Criticality Term

Criticality is a term that was introduced by Reeves [27]. This method considers the effect of the failures that can be detected by the sensors on the system. It is based on the Fussell-Vesely importance measure. The importance measure considers the failure’s contribution towards the unavailability of the system.

The criticality term is measured by subtracting the probability of system failure given that the sensor does not detect the critical failure. It is given by the following equation:

(8) $$\begin{array}{c} CR_{\left\{s\right\}}=\frac{Q_{sys}-Q_{sys}\left(q_{s}=0\right)}{Q_{sys}} \end{array}$$

where *Q_sys_* is the probability of system failure *Q_sys_*(*q_s_* = 0) is the probability of system failure given that the sensor does not detect the failure.

The value of the criticality term is 1 if a sensor can detect all the failures and 0 if it cannot. From the considered set of failures, only a few of the failures are considered critical failures and are marked as such. The critical failures are identified based on the method proposed by Konstantinidis, Katsavounis and Botsaris [28].

### 2.2. Ordered Fuzzy C-Means Clustering with PROMETHEE Algorithm

The analysis for sensor selection presented in this paper is based on the Ordered Fuzzy C-means clustering (OFCM) algorithm combined with the preference ranking organization method for enrichment evaluation (PROMETHEE) proposed by Bai et al. [29]. This method was developed to overcome the shortcoming of traditional clustering algorithms in which the clusters have little to no relation with one another, and the weight of each criterion is not considered. The classic fuzzy c-means clustering algorithm typically utilizes Euclidean norm for similarity measure between objects, which does not consider the relative importance of the criteria under consideration. The PROMETHEE method considers the difference between the criteria as well as the priority degree for each pair of objects. It is an efficient method for the pairwise comparison of a given set of alternatives. However, the algorithm alone does not provide the specifications for each type of sensor selected. This is achieved by utilizing a method called three-sieve sensor selection, proposed in [14].

Given a set of alternatives *A* = {*a*_1_, *a*_2_, …, *a*_n_} and a set of criteria *G* = {*g*_1_, *g*_2_, …, *g*_n_}, an ordered partition of *A* should satisfy the following three conditions:

- $A=U_{i=1,\;2,\;\ldots ,\;c_{i}}C_{i}$
- $\forall \;i\;\neq j:C_{i}\cap C_{j}=\emptyset$
- $C_{1}≻C_{2}≻\ldots C_{n}$

where *C_i_* denotes the *i*th order cluster and *C*_1_ is considered as the best cluster. Similar to the classic Fuzzy Clustering Method, the authors define a new objective, shown in (9):

(9) $$\begin{array}{c} \min J_{m}=\frac{\Sigma_{i=1}^{c}\Sigma_{j=1}^{n}{\left(\mu_{ij}\right)}^{m}\phi \left(a_{j}\right)-\vartheta_{i}{}^{2}\;}{c\;\min\_(1\leq i,\;j\leq c,\;i\neq j\;\vartheta_{i}-\vartheta_{j}{}^{2}}=\frac{J_{1}}{J_{2}}\; \end{array}$$

where *ϑ_i_* represents the fuzzy centroid of the *i*th order cluster, *ϕ* (*a_j_*) is the net outranking flow and $\mu_{ij}$ is the fuzzy membership. The following steps need to be taken to implement the algorithm.

**Algorithm.** OFFCATS
Determine the difference denoted as dk (*a_i_*, *a_j_*) between the evaluation of ai and *a_j_* with respect to the criterion *g_k_*:
(10)$$\begin{array}{c} d_{k}\left(a_{i},\;a_{j}\right)=g_{k}\left(a_{i}\right)-g_{k}\left(a_{j}\right) \end{array}$$
Transform the difference *dk* (*ai*, *aj*) into a single criterion using a preference function *P_k_* (*a_i_*, *a_j_*) for each criterion *g_k_*: 
(11)$$\begin{array}{c} P_{k}\left(a_{i},\;a_{j}\right)=f_{k}\left(d_{k}\left(a_{i},\;a_{j}\right)\right) \end{array}$$ where *f_k_* (.) is a monotonically non-decreasing function varying between 0 and 1, i.e., greater the value higher the preference to *a_i_* over *a_j_* based on the criterion *g_k_*.Compute the preference degree $\pi \left(a_{i},\;a_{j}\right)$ by aggregating all the single criterion preference function in the form of a weighted sum:
(12)$$\begin{array}{c} \pi \left(a_{i},\;a_{j}\right)=\Sigma_{k=1}^{s}w_{k}.P_{k}\left(a_{i},\;a_{j}\right) \end{array}$$
Calculate the positive and the negative net outranking flow:
(13)$$\phi^{+}\left(a_{i}\right)=\frac{1}{s-1}\Sigma_{x\in A\left\{a_{i}\right\}}\pi \left(a_{i},\;x\right)$$
(14)$$\phi^{-}\left(a_{i}\right)=\frac{1}{s-1}\Sigma_{x\in A\left\{a_{i}\right\}}\pi \left(a_{i},\;x\right)$$
The positive outranking flow $\phi^{+}\left(a_{i}\right)$ denotes the extent to which the alternative $a_{i}$ is preferred to the other alternatives. The larger the value, the better the alternative and vice versa when it comes to the negative outranking flow $\phi^{-}\left(a_{i}\right)$
Compute the net outranking flow $\phi \left(a_{i}\right)$ which represents the total priority of $a_{i}$ over all the other alternatives. (15)$$\phi \left(a_{i}\right)=\phi^{+}\left(a_{i}\right)-\phi^{-}\left(a_{i}\right)$$
If $\phi \left(a_{i}\right)=1$, then $a_{i}$ is the absolute best alternative; if the net outranking flow of two alternatives is the same, then both alternatives are equal.
Set c = 2 and randomly initialize $\mu_{ij}$ of $\phi \left(a_{j}\right)$ belonging to cluster *i*.
Calculate the fuzzy centroid $\vartheta_{i}$
(16)$$\vartheta_{i}=\frac{\Sigma_{j=1}^{n}{\left(\mu_{ij}\right)}^{m}\phi \left(a_{j}\right)}{\Sigma_{j=1}^{n}{\left(\mu_{ij}\right)}^{m}}$$
Rank the cluster according to the fuzzy centroid ($\vartheta_{i})$ of each cluster. For example, if $\vartheta_{i}>\vartheta_{j}$ then $C_{i}≻C_{j}$
Update $\mu_{ij}$ based on (17):
(17)$$\mu_{ij}=\frac{\sqrt[{m-1}]{\frac{1}{\phi \left(a_{j}\right)-\vartheta_{i}}}}{\Sigma_{i=1}^{c}\left(\sqrt[{m-1}]{\frac{1}{\phi \left(a_{j}\right)-\vartheta_{i}}}\right)}$$
Repeat steps 8 and 9 until the value of $J_{1}$ in (9) has only negligible changes. Calculate $J_{2}$ and $J_{m}$. Then, let *c* = *c* + 1. If c=γ (stop value), stop; otherwise return to step 6, where the stop value γ will reach optimal value at *n*/2. 


### 2.3. Two-Sieve Sensor Specification Selection Method

After selecting the type of sensors, to identify the specification of the sensors to be placed, two-sieve sensor selection is utilized. A three-step analysis method feeds into a selection tool that can be adapted to nearly any IoT system or situation. The proposed method is a modified version of the selection process that was proposed by Jones et al. [14]. Incorporating these two methods together makes it easier for the decision-maker to identify the specification of the sensor that needs to be purchased. The sensor performance data is obtained from the sensor manufacturer’s datasheet, which feeds into the three-step analysis. The results are used to populate a two-sieve selection tool, represented as a succession of color-coded matrices. Each sieve provides a simple go/no go decision for each sensor based on the constraints. The number of candidate sensors is reduced progressively. After the final matrix is obtained, the sensor with the highest aggregate score is chosen.

#### Three-Step Analysis

A relevant set of candidate sensors can be selected only after understanding the system and its fault as a whole. The three-step analysis aids in the practitioners’ effort in understanding the system.

1. *Define the parameters to be measured*: Several parameters can be measured either directly or indirectly through simple calculations. An example of such a parameter is provided by Regtien [5], where the author uses the amount of fluid in a tank which can be measured either through mass or volume of the fluid. Several parameters are measured typically simultaneously as the complexity of the system increases;
2. *Define the performance requirements for the sensors needed for each measurement parameter*: These requirements could be related to any functional attribute of the sensor such as accuracy, resolution, sensitivity, etc. A detailed list of requirements is provided in Cheng, Azarian and Pecht [7].
3. *Consider the environment in which the system will operate and the availability of the sensors*: The environmental factors affecting the sensor performance acts as a physical constraint for the measurement system as a whole. The number of sensors that are available in the market also needs to be considered.

The three-step analysis not only provides an in-depth understanding of the processes occurring within the system but also aids in the identification of parameters for measurement. The performance requirements, derived from the three-step analysis, are used in the two-sieve tool show to identify the appropriate set of sensors. A spreadsheet template utilized for sensor selection for the three-sieve sensor selection method is provided in [14]. As OFFCATS considers the criteria for sensor selection this negates the need for a three-sieve selection process. While the first will help us identify the specification of the sensor, the second sieve will help us identify the environmental and stock requirements of the sensor.

## 3. Results

To demonstrate the effectiveness and the scalability of the framework, a wind turbine gearbox is considered for the following reasons. First, the wind turbine gearbox is simultaneously the most troublesome and the most critical system in a wind turbine. Second, the gearbox failures are generic and independent of the manufacturer. Finally, downtime due to gearbox failures takes an average of 256 h to repair and 20% of the downtime is due to gearbox failures [30]. The basic faults under consideration and fault occurring rate for wind turbine gearbox with sensors used to detect the associated faults are provided in Table 3. The corresponding sensors for each failure mode are identified and the sensor selection algorithm will provide the type and number of sensors. The sensors used to detect the failures and the associated parameters are provided in Table 4.

A gearbox is used to convert the slow rotational speed of the rotor blades of around 30 rpm to acceptable rotational speeds of 1000–1800 rpm via a high-speed shaft. It typically consists of a lubrication system, a combination of planetary gears and parallel gears held in mesh with axial and radial supporting bearings. The transformation from the low-speed stage is typically done in several stages for stepwise alteration of speed. Each of the stages usually has a ratio of about 1:4–1:5. The gears in wind turbines deal with partial loads, variable speeds, and dynamic torques due to wind speed turbulence. This puts severe stress on the gears and the bearings inside the system. Due to the friction between the surfaces, small metal particles drop in the lubricant, which is a wear-out process known as micro-pitting.

The early failures and long downtime of a gearbox make the design, construction and maintenance of wind turbines a challenge to the renewable energy industry. Although designed to achieve a lifetime of 20 years, the gearbox falls short of the lifetime by 5−7 years. Due to its massive size, the repair and replacement of gearbox components are difficult to handle. The involvement of support ships and cranes in repairing offshore wind turbines creates its own set of issues. Only a few of the wind turbine failures can be rectified on site. To repair the gearbox failures, the entire sub-system needs to be removed from the turbine with significant cost and downtime. About 38% of the total cost of replacement of the components is from the gearbox. A typical gearbox replacement costs about $300K–$775K including the rental equipment and labor costs [31].

### OFCCaTS

This section illustrates how to utilize OFCM for sensor selection. The complete list of sensors that need to be selected, as well as their associated criteria, are provided in Table 5. The complete list of sensors and the associated criteria for sensor selections is provided in Table 4. The fault detection likelihood of the sensors for all the faults can be seen in Appendix A. For sensor selection, the maximum likelihood of each sensor is selected regardless of the number of faults detected by the sensors. Let *S* = {*s_j_*|*j* = 1, 2, …, *n*} be the set of sensors.

The step-by-step procedure for clustering based on Algorithm is given as follows:

1. The preference degrees $\pi \left(s_{i},\;s_{j}\right)$ are computed between each pair of sensors and then the net outranking flow $\phi \left(s_{j}\right)$ of each sensor is calculated. For each criterion, the Gaussian preference function shown in Equation (18) is utilized.

(18) $$f_{k}\left(d\right)=\left\{\begin{array}{ll} 0 & d\leq 0\\ 1-e^{-\frac{d^{2}}{2s^{2}}} & d>0 \end{array}\right.$$

where the equal weights are assigned for each preference criteria and only the parameter ‘*s*’ needs to be fixed. The value of ‘*s*’ lies between the threshold of indifference (below which there is no preference to either of the actions) and the threshold of absolute preference (above which there is a total preference to one of the two actions) shown in Table 6.

**Figure 2:**
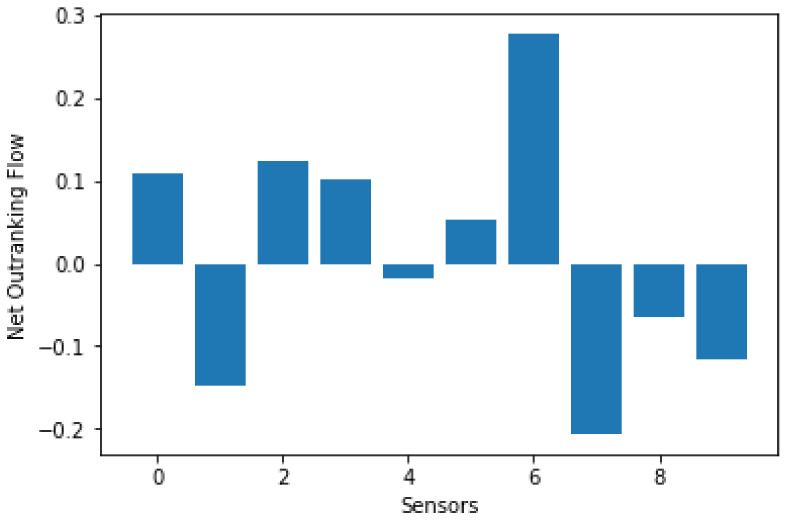
Net outranking flow for each alternative.

The preference degree of each sensor is then obtained and $\phi \left(s_{j}\right)$ is calculated using Equation (15). The net outranking flow for each sensor is shown in Figure 2 and the net outranking flow for each criterion is shown in Table 7. Then, set the number of clusters to be 2. (*c* = 2).

2. Randomly initialize the memberships of $\mu_{ij}$ of $\phi \left(s_{j}\right)$ belonging to cluster ‘*i*’.
3. The fuzzy centroid for each cluster is calculated using equation 16. Let the fuzziness parameter be set to 2.
4. Rank the clusters according to the fuzzy centroid $\vartheta_{i}$. Thus, we can obtain *C*1 and *C*2.
5. Update the value of $\mu_{ij}$ based on equation 17.
6. Repeat Steps 3 and 4 until $\left|J_{1}^{t}-J_{1}^{t-1}\right|\leq \epsilon$, where ‘*t*’ denotes the iteration and ϵ=0.000001 is the absolute difference between *J*_1_*^t^* and *J*_1_*^t^*^−1^.
7. Compute *J_m_*. Then, let *c* = *c*+1, stop when the number of clusters *c* = 10/2 ≈ 5, otherwise return to step 2.
8. Select the number of clusters with the minimum value of ‘*J_m_*’ and defuzzify the memberships

In Table 8 the membership value of each sensor for a given cluster is shown. Considering $C_{1}≻C_{2}$ the sensors belonging to cluster $C_{1}$ are selected. The next step in the process is to proceed with the two-sieve method to identify the specification for the chosen sensors. At least three different alternatives for each sensor are chosen. The specification data for each sensor are obtained through the https://www.digikey.com, website last accessed on 2 April 2021.

The two-sieve method provides the specifications of the sensors and provides a method to compare different sensor specifications with that of the system information. System information is converted to constraints and entered into the table as shown in Table 9

An example of oil particle counters from different sensor providers is shown in Table 9. In the example shown below, the color codes are keyed to scores: red = 0, yellow = 1, and green = 2. The total score in each sieve is calculated by multiplying the scores of each constraint. In the first sieve, the sensors with scores more than 0 are filtered out. In the second sieve, the scores are calculated by multiplying the scores of the first sieve with that of the total score of the second sieve, and the sensor with the maximum score is selected. In this case, the ‘Filtertechnik PC9001′ is selected.

This process is repeated for all the other types of sensors that are selected. In the two-sieve method the scores do not reflect the sensor performance in the long run nor do they indicate the superiority of any sensor over another.

## 4. Conclusions

In this paper, a sensor selection framework for designing a fault diagnostic system has been presented. A case study based on the gearbox subsystem of a wind turbine is provided to demonstrate the OFCCaTS methodology, utilizing a fuzzy clustering method with preference ranking that has been established based on the wind turbine gearbox fault history data and expert experience. The constraints and metric proposed in the graphical method proposed in this paper generate two 2D graphs. No candidates are discarded between the graphs so that analysis is performed with simultaneous comparison of multiple graphs. Further, while most sensor selection processes become unmanageably complex given a large system, the proposed process is scalable and generalizable to any size system. In contrast to the complex, and sometimes specialized approaches, the proposed sensor selection process offers a more general and easily adaptable approach.

In the case of an extremely large system, the proposed method is scalable until the OFCM step, where the two-sieve method becomes time-consuming and extremely inefficient. Future work will investigate extending the process to automate the two-sieve step of the proposed sensor selection method such that the decision-maker is provided with a list of sensors with associated specifications.

## Figures and Tables

**Table 1:** Summary of Literature Review.

Authors	Title	Key Attributes
J. Shieh, J. E. Huber, N. A. Fleck, and M. F. Ashby	The Selection of Sensors	Provides an overview of sensor performance thus graphically illustrating the sensors best suited for a given task
Schmidt and Laerhoven	How to build smart appliances?	Variables that help discriminate the contexts are identified. Sensors are selected to account for the accuracy and cost of the sensors.
Kertiou et al.	A dynamic skyline technique for a context-aware selection of the best sensors in an IoT architecture	This method can be adopted by different IoT middleware for designing relevant solutions with a high level of accuracy and minimize the search and selection time
Tjen, Smarra, and D’Innocenzo	An entropy-based sensor selection algorithm for structural damage detection	PCA is used to achieve trade-off between prediction accuracy and computational complexity
Jones et al.	A Straightforward Route to Sensor Selection for IoT Systems	It considers performance requirements, environmental constraints, and economic considerations. This method starts with an analysis of the system. The candidate sensors are assessed for specific requirements from the operators and the final decision is based on the cost of the sensors
P. A Emin, Aktan; F Necati, Catbas; Kirk A, Grimmelsman; Mesut	Development of a model health monitoring guide for major bridges	Candidate set of sensors are selected in consideration of performance characteristics, environmental constraints, and cost
Zhang and Vachtsevanos	A Methodology for Optimum Sensor Localization / Selection in Fault Diagnosis	Fault detectability metric is quantified. Cost is the modeled objective that is optimized using particle swarm optimization.
Riedel, Arroyo, and Fay	Knowledge-based selection of principle solutions for sensors and actuators based on standardized plant description and semantic concepts	The paper presents a concept based on plant description and semantic models. The function-oriented selection process is capable of considering a wider solution space as well as seamless integration of this procedure into plant workflow
Amol Kulkarni, Janis Terpenny, and Vittal Prabhu	Sensor Selection Framework for Designing Fault Diagnostics System	The key step that sets this sensor selection process apart is the utilization of constraints that are general to most engineered systems while also catering to the specific needs of each system with the integration of the two-sieve method

**Table 2:** Fault Sensor mapping matrix.

	s1	s2	s3	…	sn
*f*_1_	0	1	0	…	1
*f*_2_	1	1	0	…	0
⋮	⋮	⋮	⋮	⋮	
*f*_m_	0	0	1	…	0

**Table 3:** Faults under consideration.

System	Faults	Fault Occurrence Rate (/year)	Sensors to Detect the Faults
Gearbox	Abnormal Filter (f1)	0.0158	S5, S6, S10
	Poor quality of lubrication oil (f2)	0.0158	S3, S4, S6, S10
	Dirt (f3)	0.0126	S5, S10
	Abnormal vibration (f4)	0.0187	S1, S2, S8, S9
	Corrosion of pins (f5)	0.1051	S2, S5, S6, S8, S10
	Abrasive wear (f6)	0.0876	S2, S8, S9
	Glued (f7)	0.0021	S2, S3, S9
	Pitting (gear) (f8)	0.0114	S2, S6, S8, S10
	Pitting (gear bearing) (f9)	0.0263	S2, S6, S8, S10
	Excessive pressure (f10)	0.0088	S4
	Excessive temperature (f11)	0.0021	S3
	Gear fatigue (f12)	0.0026	S1, S2, S8, S9
	Tooth surface defects (f13)	0.0026	S10
	Gear tooth deterioration (f14)	0.0026	S2, S8
	Cracks in gears (f15)	0.0135	S2, S8
	Oil leakage (f16)	0.3504	S4, S5, S7

**Table 4:** Sensors and associated parameters for Sensor Selection.

Sensors	Failure Rate (/hour)	Proportion of Faults Detected	Sensor Value (cost/sensor)	Probability of Failure	Criticality Term
Strain sensor (S1)	3 × 10^−7^	0.043478	192.33	0.051	0.84
Vibration Sensor (S2)	4.22 × 10^−7^	0.195652	220.44	0.071	0.92
Temperature Sensors (S3)	8.5 × 10^−7^	0.065217	156.20	0.013	0.76
Pressure (S4)	5 × 10^−7^	0.065217	165.08	0.084	0.78
Flow (S5)	1.7 × 10^−5^	0.086957	97.14	0.949	0.60
Oil Debris sensor (S6)	1.852 × 10^−6^	0.108696	130.87	0.277	0.68
Level (S7)	4.3 × 10^−7^	0.021739	48.34	0.073	0.97
AE sensor (S8)	4.29 × 10^−6^	0.173913	209.95	0.528	0.89
Rotary Torque Sensor (S9)	5 × 10^−6^	0.086957	205.63	0.584	0.87
Oil Particle Counter(S10)	9 × 10^−7^	0.152174	239.35	0.146	0.84

**Table 5:** The complete list of sensors and the associated criteria for sensor selection.

Sensors	Fault Detection Likelihood Estimates	The proportion of Faults Detected	Sensor Value	Probability of Failure	Criticality Term
Strain sensor (S1)	0.85	0.043478	192.33	0.051	0.84
Vibration Sensor (S2)	0.94	0.195652	220.44	0.071	0.92
Temperature Sensors (S3)	0.826	0.065217	156.20	0.013	0.76
Pressure (S4)	0.97	0.065217	165.08	0.084	0.78
Flow (S5)	0.92	0.086957	97.14	0.949	0.60
Oil Debris sensor (S6)	0.818	0.108696	130.87	0.277	0.68
Level (S7)	0.94	0.021739	48.34	0.073	0.97
AE sensor (S8)	0.94	0.173913	209.95	0.528	0.89
Rotary Torque Sensor (S9)	0.78	0.086957	205.63	0.584	0.87
Oil Particle Counter (S10)	0.93	0.152174	239.35	0.146	0.84

**Table 6:** The preference thresholds and the weights of the five criteria.

Threshold of Indifference (*q*)	0.0409	0.229936	0.0472	0.672	0.81
Threshold of absolute preference (*p*)	0.1412	0.840427	0.4652	0.885	0.94
Weights (*w_i_*)	1/5	1/5	1/5	1/5	1/5

**Table 7:** Net outranking flow for each criterion.

Sensors	*g*_1_	*g*_2_	*g*_3_	*g*_4_	*g*_5_
Strain Sensor	0.043	0.753835	0.051	0.84	0.85
Vibration Sensor	0.196	0.901	0.071	0.92	0.94
Temperature Sensor	0.065	0.564682	0.013	0.76	0.826
Pressure Transducer	0.065	0.611172	0.084	0.78	0.97
Flow Sensor	0.087	0.255484	0.949	0.6	0.92
Oil Debris Sensor	0.109	0.432072	0.277	0.68	0.818
Level Sensor	0.022	0	0.073	0.97	0.94
AE sensor	0.174	0.846081	0.528	0.89	0.94
Rotary Torque Sensor	0.087	0.823465	0.584	0.87	0.78
Oil Particle Counter	0.152	1	0.146	0.84	0.93

**Table 8:** The membership value of each sensor for the cluster with minimum value of *J_m_*.

Sensors	Cluster 1		Cluster 2
Strain Sensor	0.613501		0.386499
Vibration Sensor	0.861319		0.138681
Temperature Sensor	0.555019		0.444981
Pressure Transducer	0.439907		0.560093
Flow Sensor	0.730557		0.269443
Oil Debris Sensor	0.360895		0.639105
Level Sensor	0.526111		0.473889
AE sensor	0.418491		0.581509
Rotary Torque Sensor	0.586528		0.413472
Oil Particle Counter	0.752694		0.247306

**Table 9:** Selection of an oil sensor using the two-sieve method.

Sieve 1 Performance Requirements		Criteria						Total
		Description	Minimum Detectable Particle Size (>4 µm)	Detects Both Ferrous and Non-Ferrous Particles	Fluid Temperature	Fluid Compatibility	Detects Humidity	
Sensors	S1	Gill Sensors & Controls 4212 OIL CONDITION SENSOR	1 µm	No	40 to 150	Hydraulic, gear, mineral, vegetable synthetic ester, semi-synthetic, polyalphaolefin, polyalkyleneglycol	No	8
	S2	SKF CMSS-ONL-1000-2	Ferrous—40 µm Non-Ferrous—135 µm	Yes	−20 to 85	Mineral, synthetic oils and water/oil emulsions	Yes	0
	S3	Filtertechnik PC9001	4 µm	Yes	0 to 70	Hydraulic and lubrication oils, mineral, synthetic (phosphate ester compatible) diesel fuels	Yes	16
	S4	Pamas S50	4 µm	No	20 to 60	Mineral and synthetic oils	No	1
Total	4	Total Score = Minimum Detectable Particle × Detects both types of particles × Fluid Temperature × Fluid Compatibility						3
**Sieve 2 Physical & Environment Requirements**		**Criteria**						**Total**
		**Description**	**Operating Temperature 29–50 °C**		**Sensor Housing Dimension Volume (mm^3^)**		**Weight (kg)**	
Sensors	S1	Gill Sensors & Controls 4212 OIL CONDITION SENSOR	Yes		727,650		0.84	32
	S2	SKF CMSS-ONL-1000-2	Yes		847,547		0.75	64
	S3	Filtertechnik PC9001	Yes		31,680,000		5	0
Total	4	Total Score = Total Score Sieve 1 × Operating Temperature × Sensor Housing Dimension × Weight						1

## Data Availability

Not applicable.

## Appendix A

**Table A1:** Sensor and fault mapping with their probability of detection.

	Strain Sensor	Vibration Sensor	Temperature Sensor	Pressure Transducer	Flow Sensor	Oil Debris Sensor	Level Sensor	AE Sensor	Rotary Torque Sensor	Oil Particle Counter
	**S1**	**S2**	**S3**	**S4**	**S5**	**S6**	**S7**	**S8**	**S9**	**S10**
**f1**	0	0	0	0	0.641	0.709	0	0	0	0.78
**f2**	0	0	0.52	0.59	0	0.82	0	0	0	0.72
**f3**	0	0	0	0	0.59	0	0	0	0	0.93
**f4**	0.72	0.77	0	0	0	0	0	0.84	0.72	0
**f5**	0	0.64	0	0	0.45	0.45	0	0.50	0	0.59
**f6**	0	0.82	0	0	0	0	0	0.94	0.63	0
**f7**	0	0.84	0.56	0	0	0	0	0	0.78	0
**f8**	0	0.64	0	0	0	0.56	0	0.8	0	0.63
**f9**	0	0.68	0	0	0	0.65	0	0.67	0	0.56
**f10**	0	0	0	0.97	0	0	0	0	0	0
**f11**	0	0	0.83	0	0	0	0	0	0	0
**f12**	0.85	0.83	0	0	0	0	0	0.84	0.54	0
**f13**	0	0	0	0	0	0	0	0	0	0.73
**f14**	0	0.94	0	0	0	0	0	0.72	0	0
**f15**	0	0.76	0	0	0	0	0	0.85	0	0
**f16**	0	0	0	0.78	0.92	0	0.94	0	0	0
